# Guillain-Barré Syndrome Following an Extended-Spectrum Beta-Lactamase Escherichia coli Urinary Tract Infection: A Case Report

**DOI:** 10.7759/cureus.19673

**Published:** 2021-11-17

**Authors:** Aaron Fischer, Juan Avila

**Affiliations:** 1 Internal Medicine, Methodist Dallas Medical Center, Dallas, USA

**Keywords:** guillain-barré syndrome, extended-spectrum beta- lactamase escherichia coli urinary tract infection, intravenous immunoglobulin, post-infection inflammatory polyradiculoneuropathy, ascending flaccid paralysis, molecular mimicry

## Abstract

Guillain-Barré syndrome (GBS) is a rare autoimmune disorder that typically develops after a respiratory or gastrointestinal infection. While *Campylobacter jejuni *is associated with approximately 30% of cases, organisms such as *Haemophilus* *influenzae*, *Mycoplasma* *pneumonia*, Epstein-Barr virus, cytomegalovirus, Zika virus, influenza virus, and hepatitis A, B, C, and E have demonstrated clinical associations to GBS. In rare instances, *Escherichia* *coli* infections have been documented as the underlying cause for GBS. Our patient, a 69-year-old female, was admitted with a two-week history of progressively worsening bilateral lower extremity weakness following diagnosis of an extended-spectrum beta-lactamase *E. coli* urinary tract infection. She was diagnosed with GBS based on acute flaccid paralysis, areflexia, and a nerve conduction velocity study showing an absent motor response in her lower extremities bilaterally. The patient subsequently underwent intravenous immunoglobulin (IVIG) treatment for five days which resulted in significant improvement in her bilateral lower extremity weakness, a response consistent with the diagnosis of GBS.

## Introduction

Guillain-Barré syndrome (GBS) is a polyradiculoneuropathy characterized by flaccid paralysis and areflexia most commonly from a post-infectious inflammatory process [1]. Since the eradication of poliomyelitis, GBS has become one of the most common causes of acute, generalized paralysis with an annual incidence of 0.75 to 2 cases per 100,000 in the population [2]. The majority of individuals with GBS have gastrointestinal or upper respiratory symptoms one to three weeks prior to the onset of neuromotor deficits [1,3]. Symptoms begin with paresthesias in the toes or fingers, which is generally followed by ascending lower extremity paralysis and areflexia over the course of a few days. In severe cases, paralysis can extend to the diaphragm, inducing respiratory failure that requires invasive ventilatory support [4,5].

The microorganism *Campylobacter jejuni* is most commonly associated with GBS, comprising about 30% of cases. However, other organisms such as Haemophilus influenzae, Mycoplasma pneumonia, Epstein-Barr virus, cytomegalovirus, Zika virus, influenza virus, SARS-CoV-2 virus, as well as hepatitis A, B, C, and E have been linked to GBS. On rare occasions, infection with Escherichia coli has also been identified as an inciting factor [1-5]. Our clinical case highlights a unique instance of an extended-spectrum beta-lactamase (ESBL) E. coli urinary tract infection inducing GBS with characteristic flaccid paralysis, areflexia, and a diagnostic nerve conduction velocity (NCV) study showing an absent motor response in her lower extremities bilaterally. This case report intends to further support the currently sparse literature available on the subject.

## Case presentation

A 69-year-old female with a past medical history of metastatic breast cancer status posttreatment with abemaciclib/fulvestrant and radiation therapy, iron deficiency anemia, diabetes mellitus type 2, hyperlipidemia and a recent left leg deep vein thrombosis status post thrombectomy and stent placement presented from an outpatient rehabilitation unit due to three days of worsening confusion as reported by her family. Approximately 5 weeks ago, the patient began experiencing acute intermittent confusion for a few days as reported by her family followed by subjective fevers. She subsequently was admitted to an outside hospital and was diagnosed with an ESBL E. coli urinary tract infection. After one week of receiving IV antibiotics, the patient was discharged with an additional one-week course of oral ciprofloxacin for treatment. During that prior hospitalization, her course was complicated by the onset of progressively worsening bilateral lower extremity weakness and paresthesias which severely limited her ability to ambulate. At that time, this complication was believed to be secondary to being in a state of sepsis due to the patient’s urinary tract infection. Consequently, she was started on ciprofloxacin and was discharged to an acute rehabilitation facility; however, she experienced little to no improvement in her strength despite being compliant with her prescribed antibiotics.

On physical examination, the patient had normal vital signs and was noted to be alert and oriented with no signs of apparent respiratory distress. No suprapubic or costovertebral angle tenderness was elicited. Her neurologic exam demonstrated significantly decreased strength of her bilateral lower extremities with preserved sensation to light touch grossly. The remainder of her exam was unremarkable. Pertinent admission laboratory data included a leukocytosis of 18,300/uL with neutrophilic predominance, normocytic anemia with a hemoglobin of 8.9 mg/dL and mean corpuscular volume of 97.1 fL, hypercalcemia of 12.9 mg/dL, mild hypoglycemia of 69 mg/dL, and decreased thyroid-stimulating hormone at 0.16 uIU/mL but normal T3 and T4 levels, normal vitamin B12, vitamin B1 and folate levels, negative blood cultures and a fairly normal urinalysis. Chest x-ray and computed tomography scan without contrast of the head showed no acute abnormalities. Of note, the patient had a brain magnetic resonance imaging (MRI) study approximately one-month prior that showed a small right frontal meningioma that appeared stable on her CT this admission. CT of the lumbar spine without contrast showed mixed lytic/sclerotic appearance of the bones suggesting metastatic disease and multilevel spondylosis without high-grade spinal canal stenosis.

**Figure 1:**
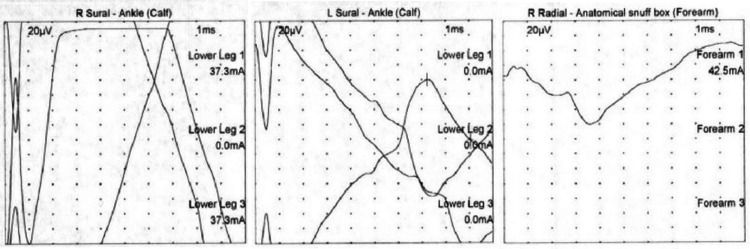
Sensory nerve conduction.

**Figure 2:**
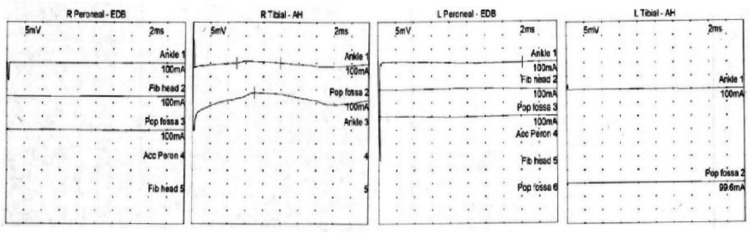
Motor nerve conduction studies revealed no response in the lower extremities. AH: abductor hallucis; EDB: extensor digitorum brevis.

**Figure 3:**
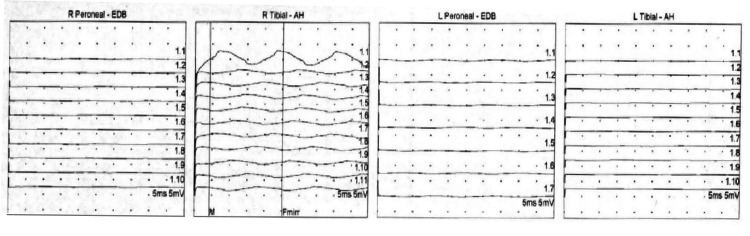
F-wave study showing the lack of reflex motor response in lower extremity muscles. AH: abductor hallucis; EDB: extensor digitorum brevis.

Given the patient's presentation of acute encephalopathy in the setting of leukocytosis and history of recent ESBL *E. coli* UTI, there was concern that the patient was inadequately treated with ciprofloxacin. Thus, she was given a one-time dose of Fosfomycin, along with IV fluids for her hypercalcemia, which improved her mental status and laboratory abnormalities. Work-up for the hypercalcemia was otherwise unremarkable and ultimately attributed to dehydration. Inpatient neurology and neurosurgery services were consulted for additional assistance with the management of acute paraparesis. No neurosurgical interventions were warranted. Additional laboratory work-up, including serum and urine protein electrophoresis, neuronal antibody (amphiphysin), CV2.1 IgG antibody and Purkinje cell/neuronal nuclear IgG antibody levels, was unremarkable. The patient underwent further imaging with MRI of the cervical, thoracic, and lumbar spine, which showed diffuse osseous metastatic lesions but no evidence of spinal cord compression or severe stenosis. A subsequent electroencephalogram demonstrated no signs of any epileptiform activity. An electromyography (EMG) study was obtained revealing complex polyphasic motor unit potentials in the right anterior tibialis, medial gastrocnemius, vastus lateralis, and tensor fascia lata muscles. Paraspinal muscles were not examined due to the patient being on anticoagulation for her history of deep vein thrombosis. Sensory nerve conduction studies were obtained and shown below (Figure 1, Table 1). Motor nerve conduction studies were obtained which showed no response in the lower extremity muscles or in the right radial study (Figure 2, Table 2). The patient also had poor F-wave responses on motor testing in her lower extremity muscles (Figure 3). The patient was treated with a five-day course of intravenous immunoglobulin (IVIG) for suspected GBS. This resulted insignificant improvement in her lower extremity strength bilaterally. At the end of her hospital course, she was discharged to a skilled nursing facility for further care. 

**Table 1:** Sensory nerve conduction.

Nerve/sites	Rec site	Peak lat (ms)	PP Amp (µV)	Segments
Left sural – ankle (calf)				
Lower leg	Ankle			Lower leg – ankle
Lower leg	Ankle			Lower leg – lower leg
Lower leg	Ankle	7.24	81.9	Lower leg – lower leg

**Table 2:** Motor nerve conduction studies revealed no response in the lower extremities. AH: abductor hallucis; EDB: extensor digitorum brevis.

Nerve/sites	Muscle	Latency (ms)	Amplitude (mV)	Relative amplitude (%)	Duration (ms)	Segments	Distance (mm)	Lat Diff (ms)
Right Tibial – AH								
Ankle	AH	5.00	0.7	100	4.95	Ankle – AH	400	
Popliteal fossa	AH	6.98				Popliteal fossa – Ankle		1.98
Left Peroneal – EDB								
Ankle	EDB	16.15	0.3	100		Ankle – EDB	80	
Fibular head	EDB					Fibular head – Ankle		
Popliteal fossa	EDB					Popliteal fossa – Fibular head		

## Discussion

This case report illustrates the development of acute paraparesis, most consistent with the diagnosis of GBS, after an ESBL *E. coli* urinary tract infection. Two-thirds of GBS patients have antecedent respiratory or gastrointestinal symptoms which our patient denied.

Following a Campylobacter infection, IgA or IgG antibodies are produced against the bacterial cell wall and, in some cases, cross-react with various epitopes of human nerve cell gangliosides [6]. The four types of gangliosides targeted by these autoantibodies are GM1, GD1a, GT1a, and GQ1b. GBS develops as a result of molecular mimicry between human GM1 ganglioside and the lipooligosaccharide found on the cell wall surface of *C. jejuni*. However, the majority of individuals with these autoantibodies do not develop GBS [7,8].

*Escherichia coli* is an enteric Gram-negative rod that frequently causes urinary tract infections. *E. coli* contains lipopolysaccharide (LPS) on its outer capsule, as do all other gram-negative bacilli; however, studies have not demonstrated homology between E. coli LPS and the GM1 ganglioside. Documented cases of GBS patients with positive IgG antibodies to gangliosides on neurons result in low amplitude measurements for the compound muscle action potentials as well as normal to decreased nerve conduction velocities [1,2]. Only a few cases of preceding *E. coli* urinary tract infections in GBS patients have been observed, with a couple presenting as recurrent GBS. Acute motor neuronal neuropathy was seen in most of these patients with positive antibodies to the GM1 and GD1a gangliosides [1,2].

Our patient met the criteria of GBS with the clinical picture of rapidly developing paraparesis, areflexia, and nerve conduction studies showing severely reduced amplitudes with lack of motor responses in bilateral lower extremities. Given GBS is a diagnosis of exclusion, the primary attempt of our in-depth workup was to exclude all other likely diagnoses that would explain her clinical presentation. Our patient's central nervous system imaging and serum studies evaluating paraneoplastic etiologies were all negative. There have been studies showing breast cancer inducing neurological dysfunction via a paraneoplastic mechanism with the generation of autoantibodies such as Anti Hu, Ri, etc. However, these syndromes are generally associated with cerebellar dysfunction as the primary neurological issue, which our patient did not present with [9,10]. Anti Ri itself has been known to not directly affect the peripheral nervous system, which was clearly identified in our patient’s nerve conduction studies [10]. Our patient was documented to have negative paraneoplastic studies that included neuronal antibody (amphiphysin), CV2.1 IgG antibody and Purkinje cell/neuronal nuclear IgG antibody levels thus, making it very unlikely for her to have had a paraneoplastic syndrome secondary to a breast cancer etiology. Other differential diagnoses can be postulated such as our patient having acute lower extremity sensorimotor polyneuropathy secondary to the patient's history of type 2 diabetes or her past infusions of chemotherapy as explanations for her clinical presentation; however, these generally would not have had resolution of her symptoms after IVIG treatment. It could also be possible that our patient may have had an alternative autoimmune illness from an undiagnosed paraneoplastic syndrome; however, the temporal relationship between her urinary tract infection and the onset of her neurological symptoms one to two weeks after, point towards this as the most reasonable cause and is consistent with the chronological events of GBS known in the literature [3]. The objective findings supportive of GBS itself are the presence of her lower extremity areflexia, lower extremity weakness in the setting of absent motor responses on nerve conduction velocity studies of her lower extremities and right radial study as well as improvement after IVIG treatment. 

The two primary treatment options for GBS are plasmapheresis and (IVIG). Plasmapheresis reduces the autoimmune response against the patient’s nervous system by filtering out antibodies from the serum, whereas IVIG inhibits the autoantibodies directly. Plasmapheresis is most effective when used within four weeks after the initiation of symptoms. IVIG has proven to be just as efficacious when started within two weeks of symptom onset [11,12]. However, IVIG is generally used initially due to its ease of administration and lower complication risk profile [13]. Steroids have not been shown to be beneficial and some studies have indicated an association with longer recovery times [14]. Lastly, physical therapy has been shown to be a beneficial component in ensuring patients with GBS return to their baseline level of strength [15,16].

## Conclusions

This 69-year-old female’s acute flaccid paralysis, areflexia, and her absent motor response on nerve conduction velocity studies after the onset of an ESBL *E. coli* urinary tract infection points towards this as the most likely etiology of her GBS. The resolution of our patient’s neuropathy with five days of IVIG treatment indicates further evidence of the diagnosis of GBS itself and illustrates a reasonable treatment option for future patients who develop this complication of an ESBL *E. coli* urinary tract infection.
